# Supplementary material for: Integrated single-cell whole genome sequencing and spatial transcriptomics reveal latent intra-tumoral heterogeneity in ovarian cancer
Source: bioRxiv. 2025 Oct 9:2025.10.08.676897. Preprint. [Version 1] doi: 10.1101/2025.10.08.676897 (PMC12632484; doi:10.1101/2025.10.08.676897)
Supplement: Supplement 1 [file NIHPP2025.10.08.676897v1-supplement-1.pdf]

bioRxiv preprint doi: https://doi.org/10.1101/2025.10.08.676897; this version posted October 9, 2025. The copyright holder for this preprint (which was not certified by peer review) is the author/funder, who has granted bioRxiv a license to display the preprint in perpetuity. It is made available under aCC-BY 4.0 International license.

Supplemental Figure 1 – Clustering of scDNA copy number data

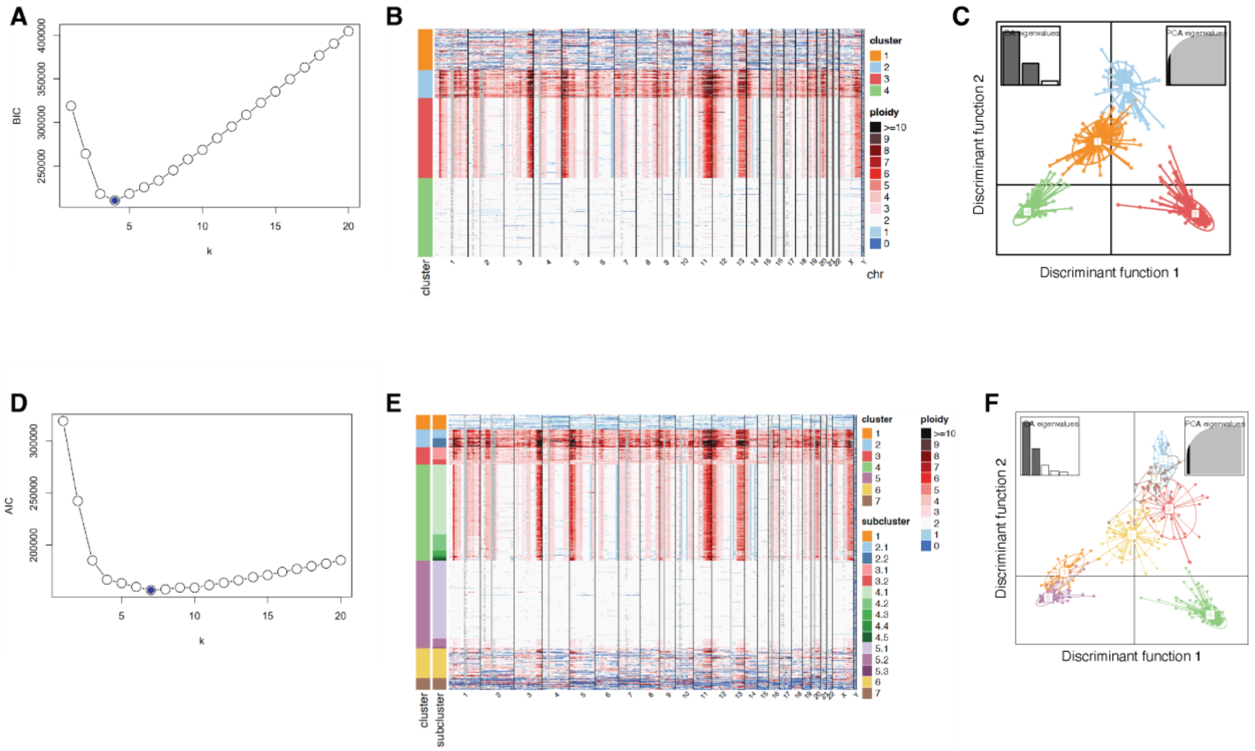

(A) Bayesian Information Criterion (BIC) of clustering solutions up to k = 20 for sample OV440. (B) Single cell copy number heatmap reflecting clustering of OV440 based on k = 4, as informed by BIC. (C) Discriminant analysis of principal components applied to k = 4 clusters of OV440. (D) Akaike Information Criterion (AIC) of clustering solutions up to k = 20 for sample OV440. (E) Single cell copy number heatmap reflecting clustering of OV440 based on k = 7, as informed by AIC. Clusters 1, 6, and 7 were excluded from further analysis due to degradation of DNA. (F) Discriminant analysis of principal components applied to k = 7 clusters of OV440.

bioRxiv preprint doi: https://doi.org/10.1101/2025.10.08.676897; this version posted October 9, 2025. The copyright holder for this preprint (which was not certified by peer review) is the author/funder, who has granted bioRxiv a license to display the preprint in perpetuity. It is made available under aCC-BY 4.0 International license.

Supplemental Figure 2 – Copy number inferred from OV440 ST data

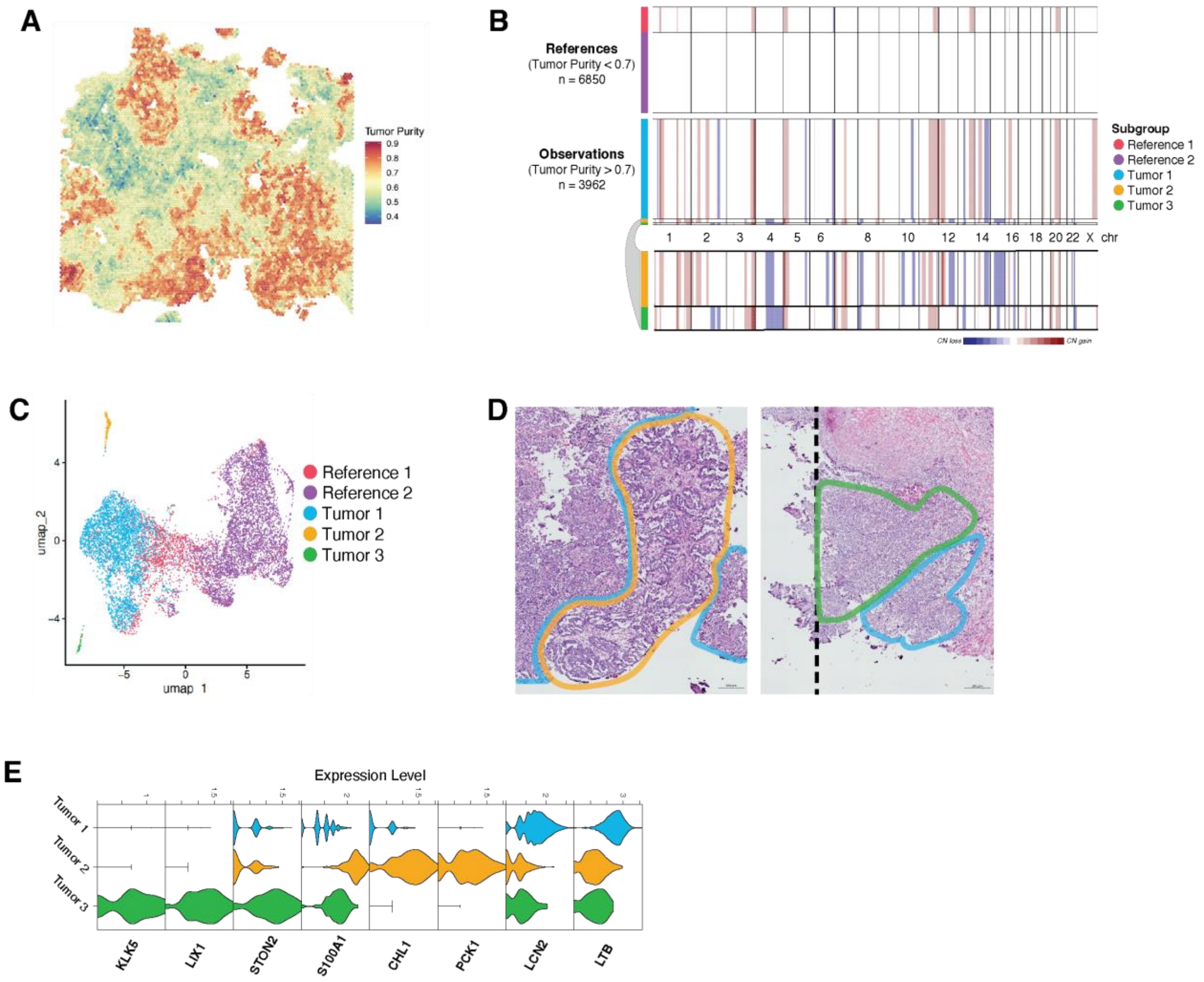

(A) Tumor purity as determined by ESTIMATE, which was used to determine reference and observation annotations for inferCNV. (B) Full inferCNV results for OV440. Subclusters were determined by Leiden clustering and are annotated on the left bar. HMM-based copy number prediction was performed at the subcluster level. Tumors 2 and 3 are expanded beneath the plot for clarity. (C) Subgroups identified in (C) mapped in low-dimensional space. Reference 2 was determined to likely comprise tumor-diploid mixtures at the tumor-stroma interface. (D) Magnified regions corresponding to the tumor subgroups identified. Tumor 2 displays a papillary growth pattern, tumor 3 displays a micropapillary growth pattern, and tumor 1 displays both micropapillary and solid growth patterns. Scale bar represents 200 microns. (E) Log-transformed gene expression of select genes differentially expressed between clusters.

bioRxiv preprint doi: https://doi.org/10.1101/2025.10.08.676897; this version posted October 9, 2025. The copyright holder for this preprint (which was not certified by peer review) is the author/funder, who has granted bioRxiv a license to display the preprint in perpetuity. It is made available under aCC-BY 4.0 International license.

Supplemental Figure 3 – TP53 mutations in HGSOC samples

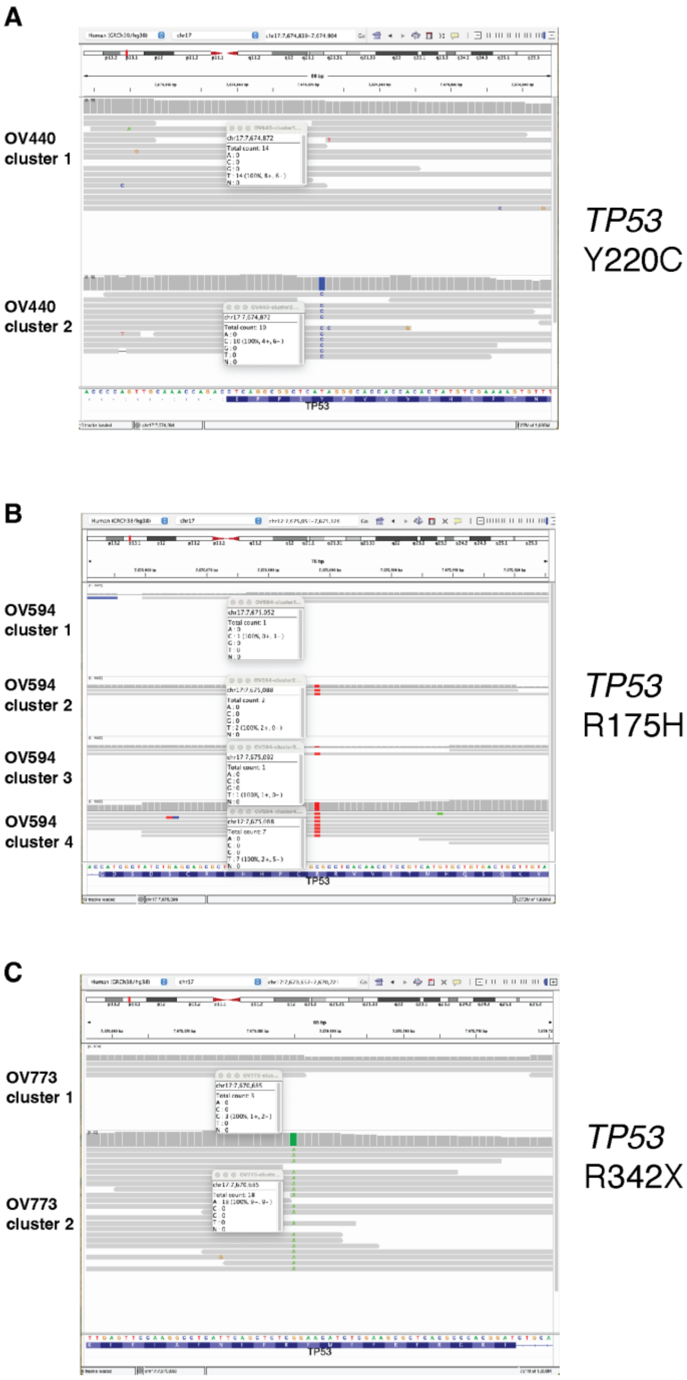

The clonal *TP53* mutations in samples (A) OV440, (B) OV594, and (C) OV773.

bioRxiv preprint doi: https://doi.org/10.1101/2025.10.08.676897; this version posted October 9, 2025. The copyright holder for this preprint (which was not certified by peer review) is the author/funder, who has granted bioRxiv a license to display the preprint in perpetuity. It is made available under aCC-BY 4.0 International license.

Supplemental Figure 4 – OV150 somatic KRAS mutation

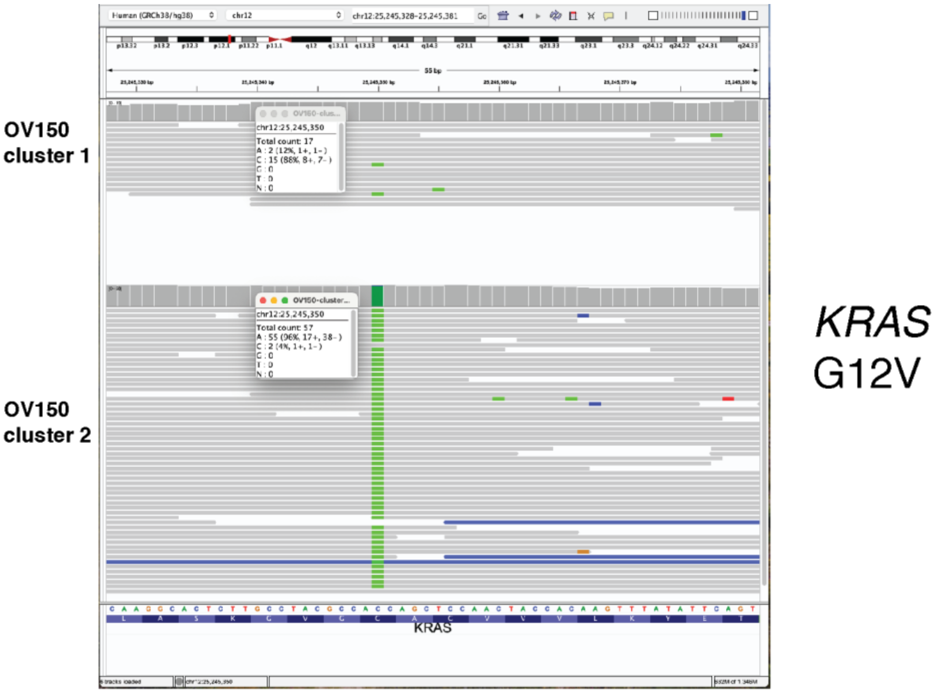

The clonal *KRAS* mutations in OV150 visualized with IGV. The two reads in cluster 1 with the mutation belong to cell 570, which is a pseudodiploid cell described in Figure 4.

bioRxiv preprint doi: https://doi.org/10.1101/2025.10.08.676897; this version posted October 9, 2025. The copyright holder for this preprint (which was not certified by peer review) is the author/funder, who has granted bioRxiv a license to display the preprint in perpetuity. It is made available under aCC-BY 4.0 International license.

Supplemental Figure 5 – Chromosome 17 LOH in OV440

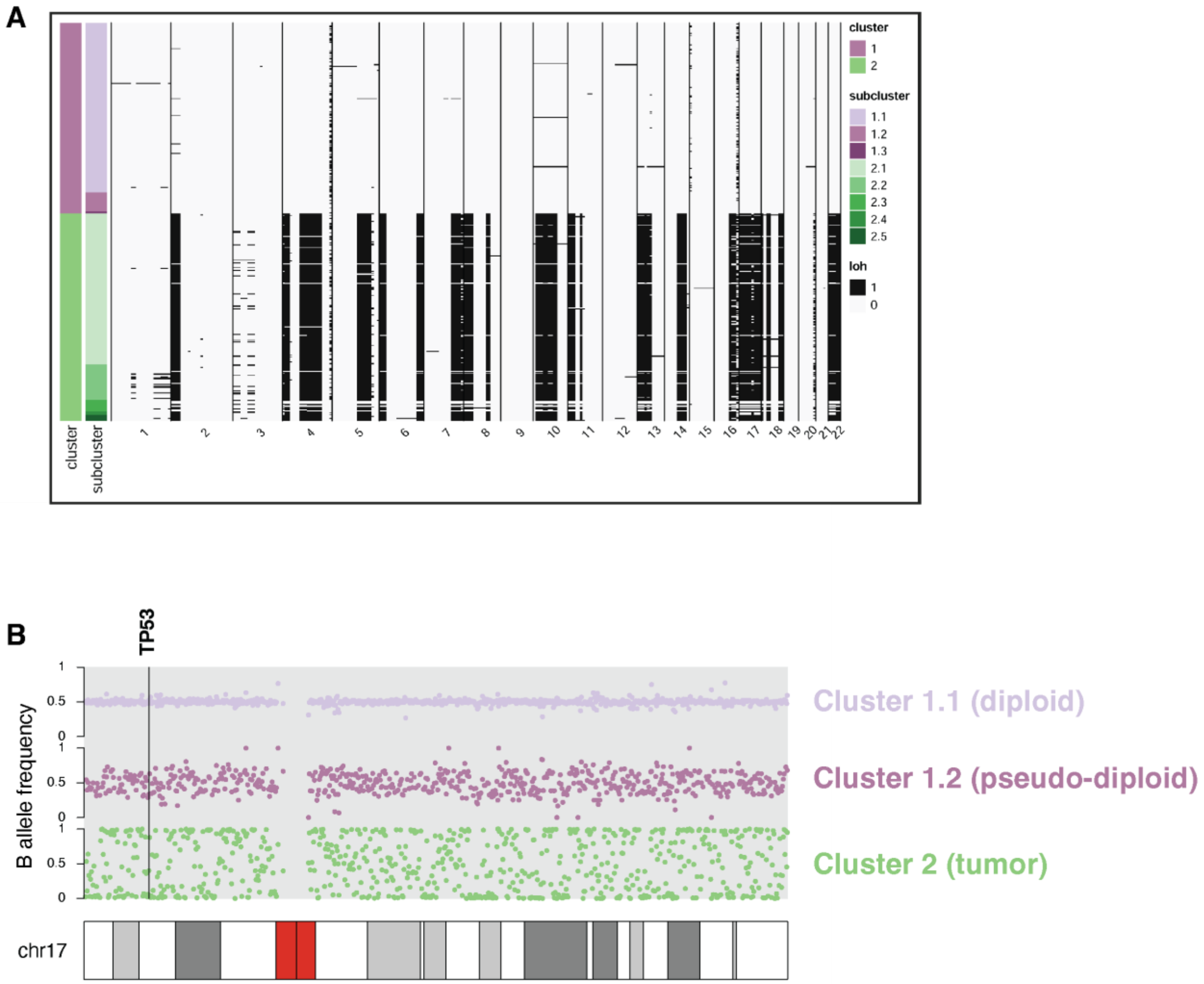

(A) Loss of heterozygosity in sample OV440 inferred from single-cell allele specific copy number determined by CHISEL. Black shading represents LOH. (B) B allele frequencies across chromosome 17 for clusters of OV440.

bioRxiv preprint doi: https://doi.org/10.1101/2025.10.08.676897; this version posted October 9, 2025. The copyright holder for this preprint (which was not certified by peer review) is the author/funder, who has granted bioRxiv a license to display the preprint in perpetuity. It is made available under aCC-BY 4.0 International license.

Supplemental Figure 6 – Evolution of OV511 subcluster 2.4

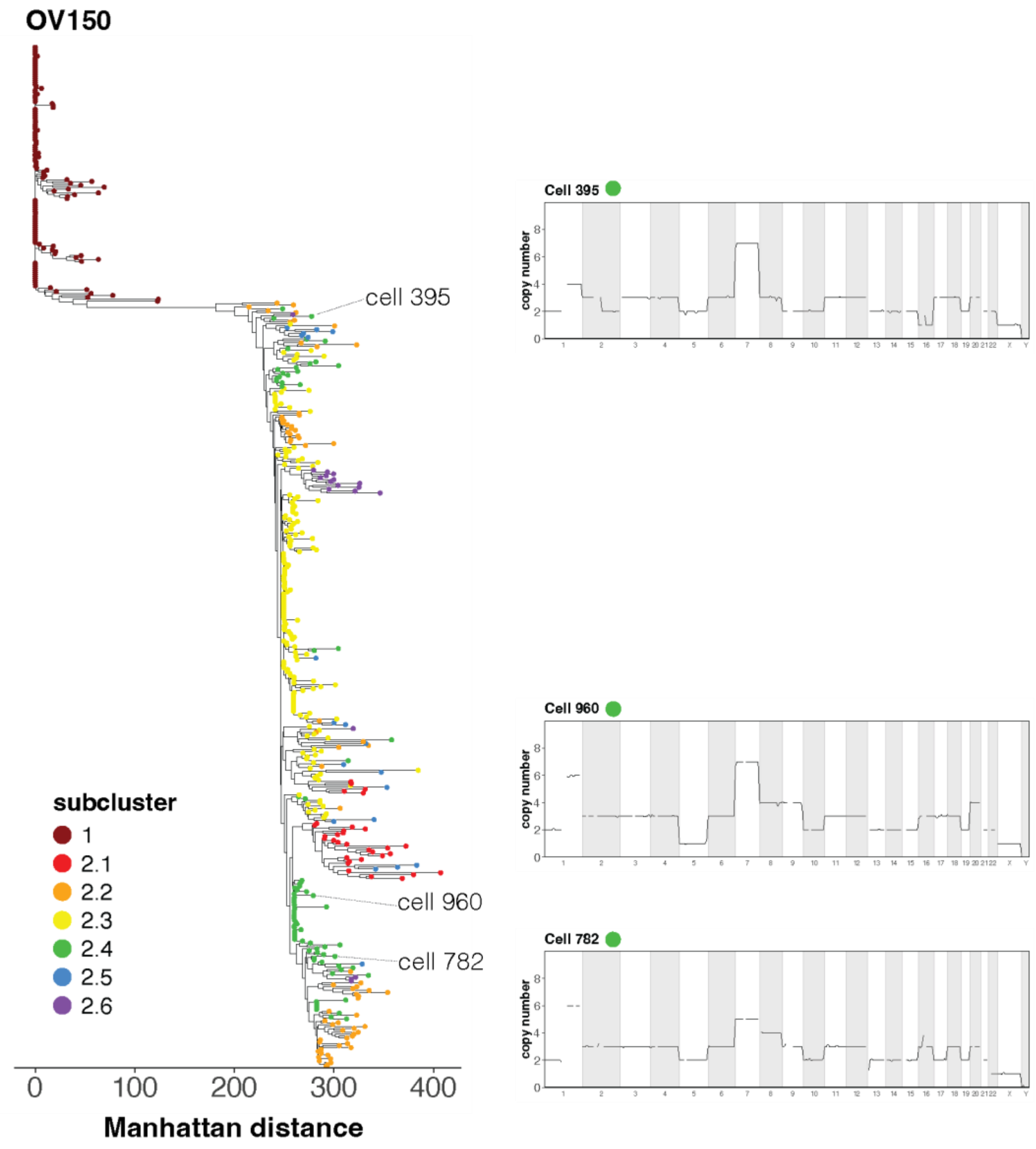

Single cell phylogeny of OV150 (also presented in Figure 4) annotated with three representative cells from subcluster 2.4 and their copy number plots.

bioRxiv preprint doi: https://doi.org/10.1101/2025.10.08.676897; this version posted October 9, 2025. The copyright holder for this preprint (which was not certified by peer review) is the author/funder, who has granted bioRxiv a license to display the preprint in perpetuity. It is made available under aCC-BY 4.0 International license.

Supplemental Figure 7 – NRF1 and NFE2L2 expression in OV594

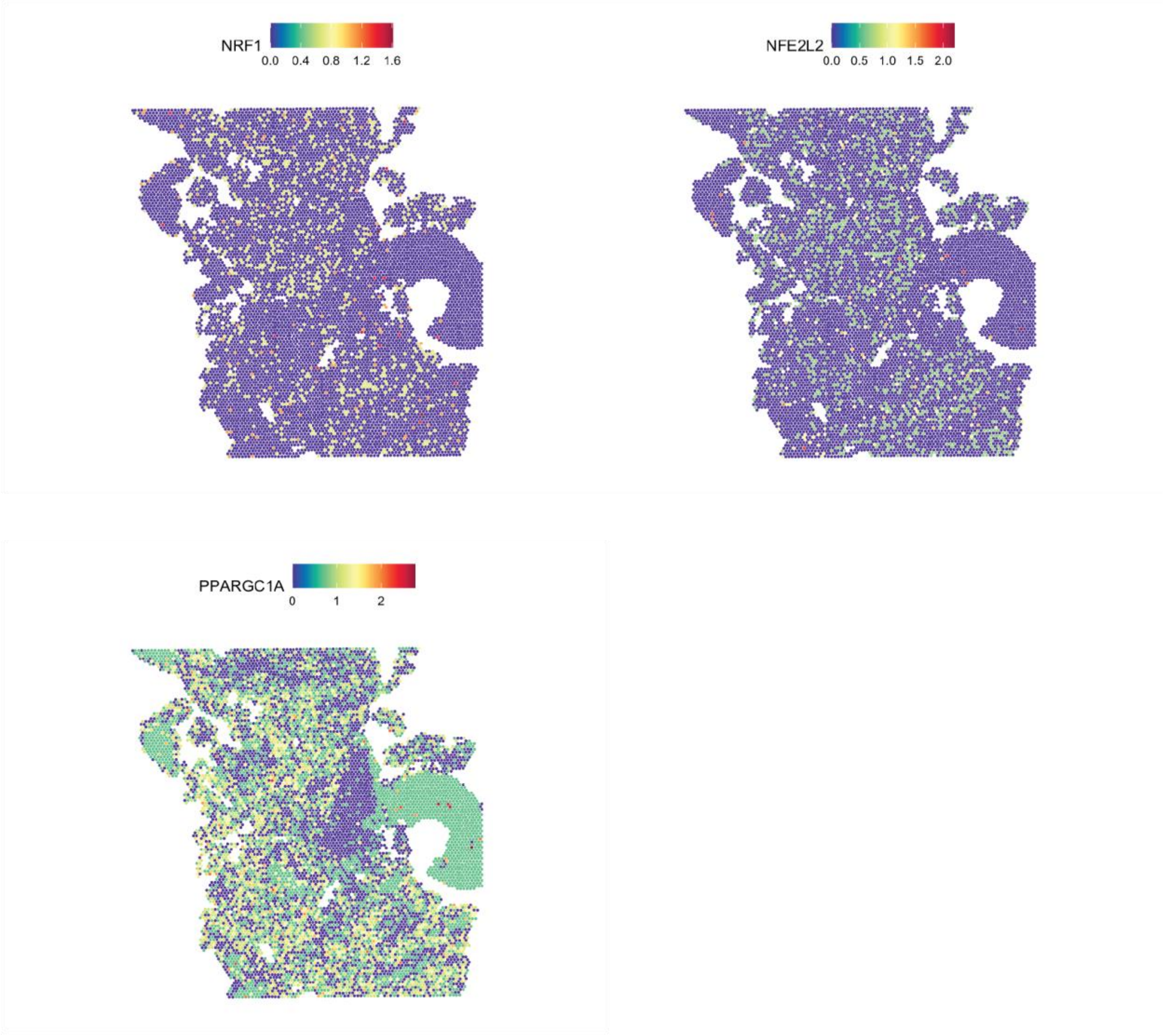

Spatially mapped log-transformed gene expression of *NRF1*, *NFE2L2*, and *PPARGC1A* in sample OV594.

bioRxiv preprint doi: https://doi.org/10.1101/2025.10.08.676897; this version posted October 9, 2025. The copyright holder for this preprint (which was not certified by peer review) is the author/funder, who has granted bioRxiv a license to display the preprint in perpetuity. It is made available under aCC-BY 4.0 International license.

Supplemental Figure 8 – Shared copy number events in OV511 clones

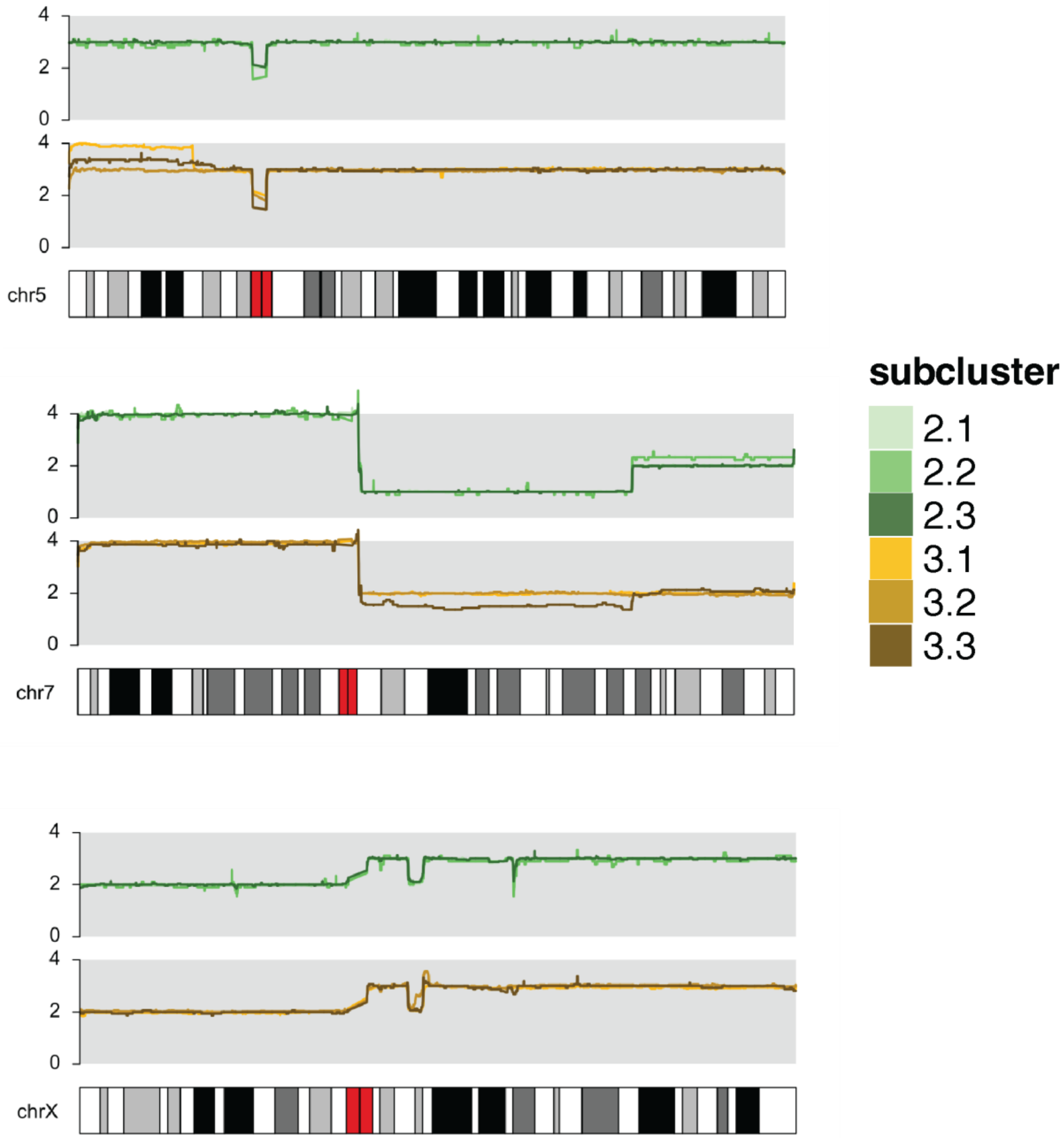

Average copy number line plots for sample OV511, at three representative shared copy number alterations. Copy number is displayed on the y-axis and plotted at 20Kb resolution.

bioRxiv preprint doi: https://doi.org/10.1101/2025.10.08.676897; this version posted October 9, 2025. The copyright holder for this preprint (which was not certified by peer review) is the author/funder, who has granted bioRxiv a license to display the preprint in perpetuity. It is made available under aCC-BY 4.0 International license.

Supplemental Figure 9 – OV511 somatic variants

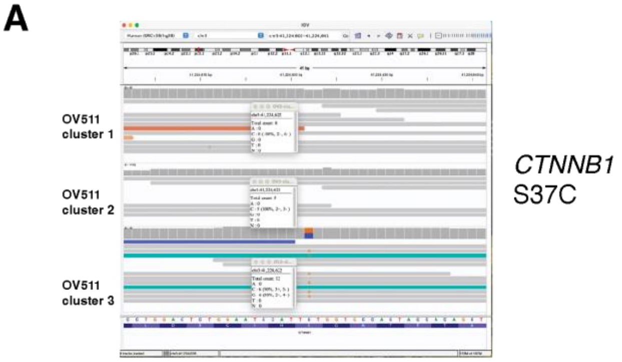

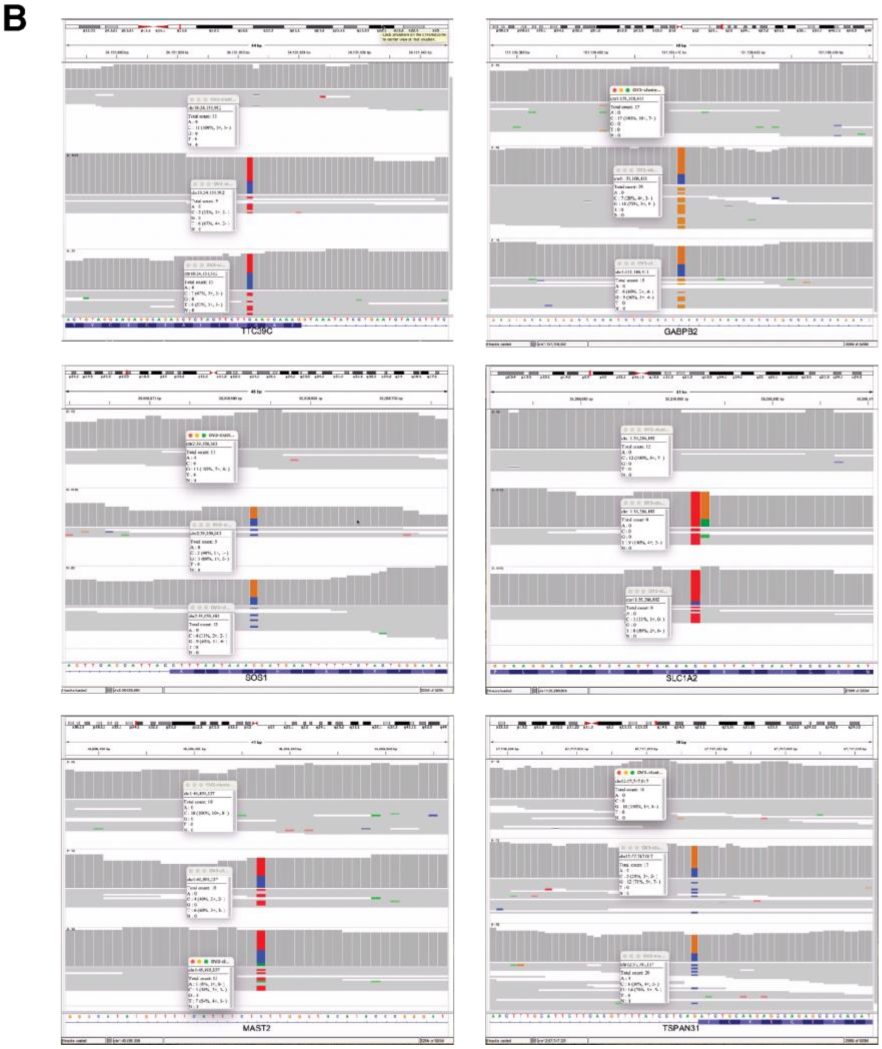

(A) The putative *CTNNB1* oncogenic driver mutation in sample OV511. (B) Select somatic passenger mutations in sample OV511.

bioRxiv preprint doi: https://doi.org/10.1101/2025.10.08.676897; this version posted October 9, 2025. The copyright holder for this preprint (which was not certified by peer review) is the author/funder, who has granted bioRxiv a license to display the preprint in perpetuity. It is made available under aCC-BY 4.0 International license.

Supplemental Figure 10 – LOH at CTNNB1 SNPs

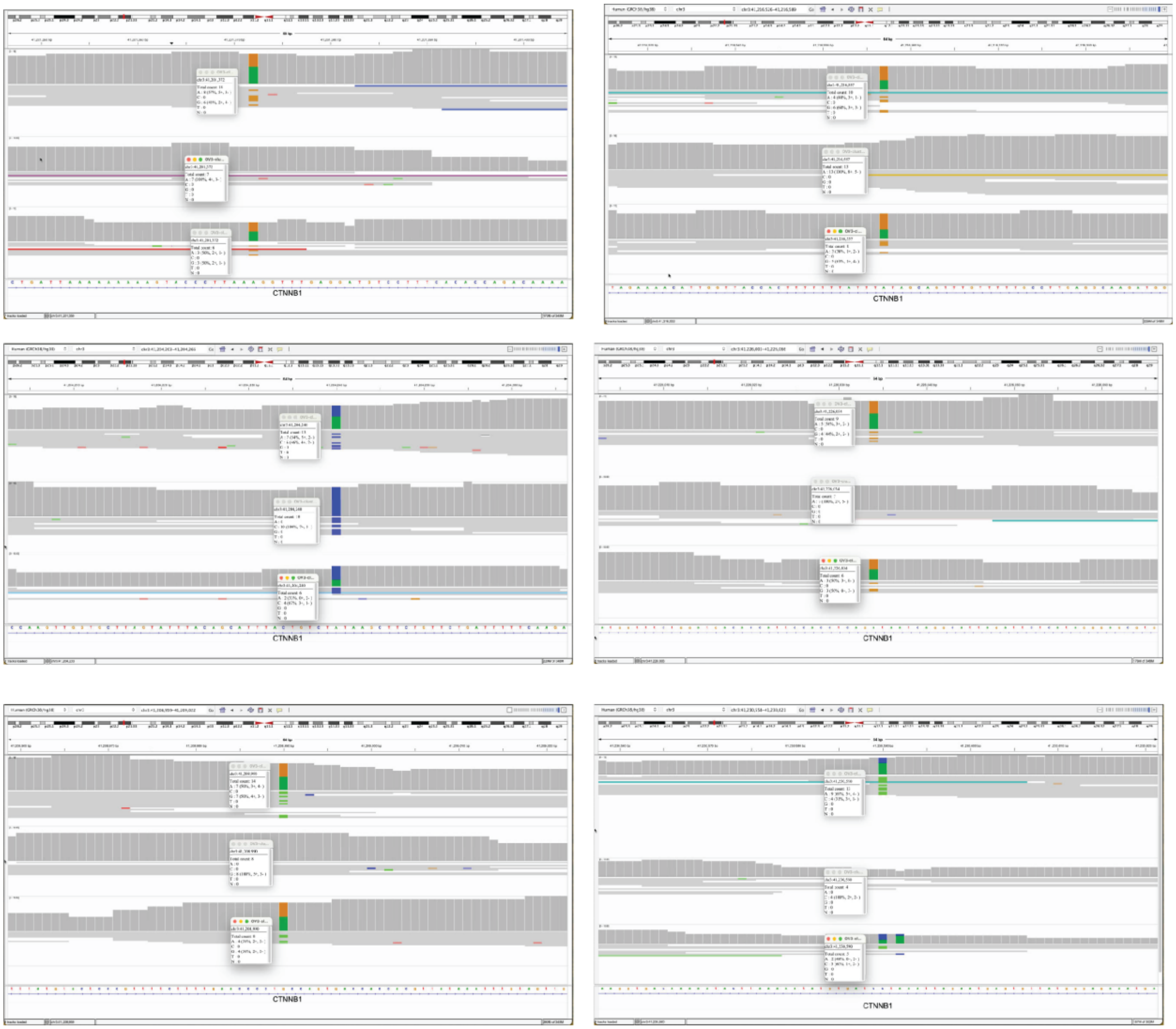

Heterozygous germline SNPs within *CTNNB1* support LOH in cluster 2 of OV511. Tracks are in order from top to bottom: cluster 1, cluster 2, cluster 3

bioRxiv preprint doi: https://doi.org/10.1101/2025.10.08.676897; this version posted October 9, 2025. The copyright holder for this preprint (which was not certified by peer review) is the author/funder, who has granted bioRxiv a license to display the preprint in perpetuity. It is made available under aCC-BY 4.0 International license.

Supplemental Figure 11 – Positional gene set enrichment in OV511

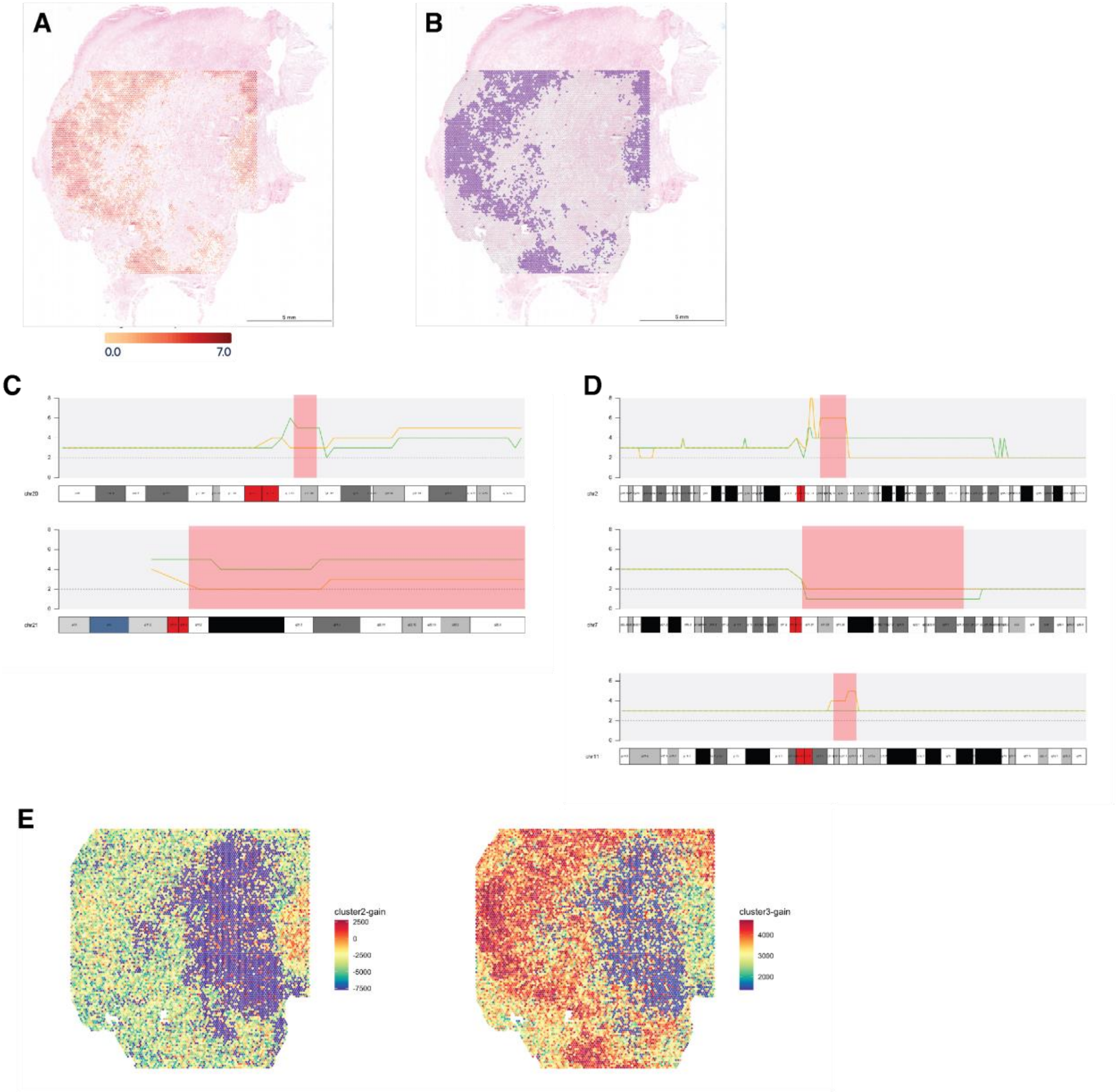

(A) Aggregate expression of *EPCAM*, *KRT7*, and *KRT8* in OV511. Scale bar represents log2 of the summed expression. (B) Spots passing the filtering threshold of log2 summed expression > 1.5. We consider these spots to be comprised mostly of epithelial cells. (C) Regions of the genome utilized in the positional gene set for cluster 2. All unique canonical genes in these regions were exported from UCSC genome browser. (D) Regions of the genome utilized in the positional gene set for cluster 3. (E) Spatially mapped ssGSEA enrichment scores corresponding to the custom positional gene sets for each cluster. Spots passing epithelial filtering (B) were assigned to cluster 2 if the cluster 2-gain score was greater than 2500, and to cluster 3 if the cluster3-gain score was greater than 3000.

bioRxiv preprint doi: https://doi.org/10.1101/2025.10.08.676897; this version posted October 9, 2025. The copyright holder for this preprint (which was not certified by peer review) is the author/funder, who has granted bioRxiv a license to display the preprint in perpetuity. It is made available under aCC-BY 4.0 International license.

Supplemental figure 12 – Histology of OV511 clones

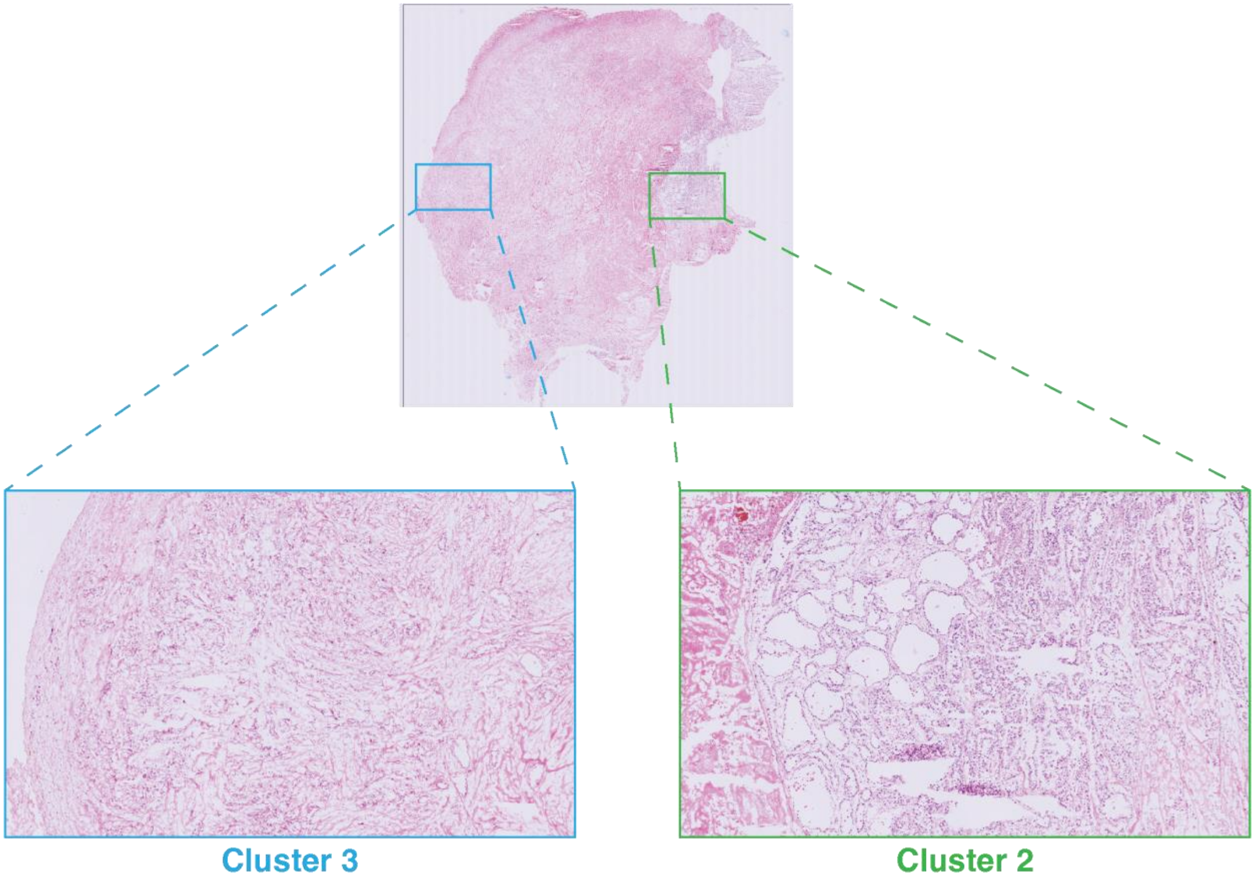

Magnified H&E-stained tissue from sample OV511. The indicated regions were determined to correspond to the labeled clusters by positional gene set enrichment.
